# Frequency of occupational health hazards and factors responsible among the waste handlers at the tertiary care hospitals of Karachi

**DOI:** 10.12669/pjms.40.7.9113

**Published:** 2024-08

**Authors:** Eshwar Das, Shiraz Shaikh, Umm-e-Rabab, Dileep Kumar

**Affiliations:** 1 Eshwar Das, MSPH. Lecturer, College of Nursing NICH, Karachi, Pakistan; 2 Dr. Shiraz Shaikh, FCPS. Associate Professor, APPNA Institute of Public Health, Jinnah Sindh Medical University, Karachi, Pakistan; 3 Dr. Umm-e-Rabab, MPH. Assistant Professor, Community Medicine, Liaquat National Hospital & Medical College, Karachi, Pakistan; 4 Dileep Kumar, MSN. Lecturer, College of Nursing Sukkar, Pakistan

**Keywords:** Hospital waste handlers (HWHs), occupational health hazards, Needle stick injury (NSI), Hospital Waste Management (HWM)

## Abstract

**Background & Objectives:**

Hospital waste handlers (HWHs) are in contact with contaminated waste that put them at risk for occupational health hazards. The objective of the study was to determine the frequency of occupational health hazards and identify factors contributing to them among the HWHs at tertiary care hospitals of Karachi

**Methods:**

A cross sectional survey was conducted from January 2021 till June 2022 on 417 conveniently selected HWHs of the public and private tertiary care hospitals of the Karachi including three Public sector hospitals (Civil Hospital Karachi, National Institute of Child Health, Jinnah Post Graduate Medical Center) and five private sector hospitals (Sohail University Hospital, Darulsehat Hospital, Kharadar General Hospital, Patel Hospital and Hamdard University Hospital) using a structured questionnaire. Chi Square test was applied to determine the differences in occurrence of different hazardous outcomes (Needle stick injury, Sharp Injury, Eye Symptoms, Skin symptoms, Cough) between different groups of age, gender, type of hospitals and status of being trained in Hospital Waste Management (HWM).

**Results:**

Around half of the HWHs (52.6%) labeled the bins of the waste according to their level of hazard. Only 17.9% disinfected the infected waste. The proportion of participants who experienced needle stick and sharp injury in the last six months was 16.3% and 15.8% respectively. Majority of them used disposable gloves (95.7%) and face masks (94.3%). One thirds had access to aprons while only 10.5% had access to protective shoes at their work place. HWHs of private sector were significantly less likely to experience Needle stick injuries, skin symptoms, cough, breathing difficulty and throat burning.

**Conclusion:**

The HWM practices in tertiary care hospitals of Karachi is far from being satisfactory. HWHs must be trained and monitored for safe disposal of waste

## INTRODUCTION

Biomedical waste may be contaminated with sharps, biological, chemical, pathological, cytotoxic agents and body parts which increases the risk of health hazards.^1^ If the contaminated waste is disposed without proper treatment, it may predispose health care workers, patients, other people and hospital waste handlers (HWHs) to risk for infections and other health problems such as: respiratory, skin, gastro-intestinal problems, lethal blood infection and injuries.^2^,^3^

The hazardous waste further can be classified into sharps (needles, knife, scalpels, broken glass), chemical waste (disinfectants, solvents, laboratory reagents), pathological waste (body tissues, unused blood products, organs), infectious waste (contaminated body fluids), pharmaceutical waste (medicines) and cytotoxic waste (anti -neoplastic agents).^4^

Hospital waste handlers (HWHs) are particularly at high risk of consequences of poor hospital waste management (HWM) practices.^5^ Inadequate training, low vaccination status for blood borne disease, lack of use of personal protective equipment (PPE) during handling and poor techniques of disposing contaminated waste by the health team members leads to increase in the risk for occupational hazards among them.^6^ The danger of getting exposed to infections is prominent because of decreased observance of the PPEs and low adherence to HWM guidelines.^7^

In developing countries like Pakistan, Segregation of waste is not properly followed in the health facilities and the utilization of PPE in these organizations is also unsatisfactory.^8^ The hazardous waste generation proportion at the teaching hospitals of the Pakistan is between 12% - 42%.^9^ The Karachi is a mega city of the Pakistan, majority of inhabitants seek health care from major tertiary care hospitals of the Karachi. All of them generate a huge quantity of the waste comprising of the sharps, solids and liquids. It has been observed that HWHs of the Karachi don’t follow the adequate protective measures nor dispose waste in the standardized way. This study was conducted to determine the magnitude of this problem on which scarce data is available. As per literature review no such study has been conducted in Pakistan for determining the occupational hazards among this high risk group although some research work has been done to determine the incidence of Needle stick injuries (NSIs) in the health care professionals working at the tertiary care hospitals. The aim of this study was to identify the specific interventions required to protect the HWHs from occupational hazards.

## METHODS

This was a cross sectional survey among the HWHs working in three public sector hospitals (Civil Hospital Karachi, National Institute of Child Health, Jinnah Post Graduate Medical Center) and five private sector hospitals (Sohail University Hospiyal, Darulsehat Hospital, Kharadar General Hospital, Patel Hospital and Hamdard University Hospital) at the Karachi. The study was conducted from Jan 2021 till Jun 2022. Sample Size was calculated on Open Epi version 3. Anticipated frequencies for occupational health hazards including NSIs (66.0% 55.8%, 39.19%, 7.1%), Respiratory problems (62.3%), Skin Problems (30%), eye problems (36.3%) were used. At confidence level of 95% and bound on error of 5% the highest sample size (n = 379) came at frequency of 55 .8%. After adjustment for 10% non-response, sample size was increased to 417.

A convenient sampling technique was adopted to enroll the participants. Equal number of HWHs were approached from public and private hospitals and those who consented to participate were interviewed.

A questionnaire was initially developed by literature review. The tool comprised of sections on socio – demographic information, exposure to health hazards and safety precautions adopted by HWHs. The tool was reviewed by two public health and one research expert. After getting permission from the approached health care settings, the list and schedule of the HWHs were obtained from the HR department or their heads. The managers of the few organizations organized their workers into groups to facilitate the data collection process. Data was collected by volunteer BSc Nursing students. The training sessions were arranged by principal investigator for them regarding data collection process. Tool was translated into Urdu and was used on those participants who could not read and write English. The data collectors were supervised on field by the Principal Investigator and forms were checked for completeness on daily basis as the authenticity of the data could be maintained.

All categorical variables were summarized in frequencies and percentages; whereas the quantitative variable also computed into categories. The outcomes were compared between different groups including age, gender, educational status, type of hospital, department, working experience and status of training on hospital waste management using Chi – Square Test. P – value of < 0.05 was considered significant. Data was entered and analyzed by using SPSS version 20.

### Ethical Approval

It was obtained from Institutional review board Jinnah Sindh Medical University with reference no. JSMU/IRB/2020/369.

## RESULTS

Data of all 418 participants was analyzed as there was no missing information in any form. The descriptive characteristics of the participants is shown in Table-I. More than half of the participants (54.5%) were in middle age group of 30-49 years while around one third (32%) were in the young age group of 18-29 years. More than two thirds were male (71.1%). Almost half of the participants were illiterate (47.8%). More than half (56%) of the participants were not trained for waste disposal management.

**Table-I:** Descriptive characteristics of the study participants (n=418).

Variable	F (%)
***Age***	
18 – 29	134 (32%)
30 – 49	228 (54.5%)
50 +	56 (13.5%)
***Gender***	
Male	297 (71.1%)
Female	121 (28.9%)
***Educational Status***	
Illiterate	200 (47.8%)
Literate	218 (52.2%)
***Working Experience***	
1-5 Years	245 (58.6%)
6 and above	173 (41.4%)
***Department/Placement Area***	
Wards or Intensive Care unit	287 (68.7%)
Outpatient Department or Office	131 (31.3%)
***Type of the Hospital***	
Public	209 (50%)
Private	209 (50%)
***Received Training for Waste Disposal***	
Yes	184 (44%)
No	234 (56%)

Hospital waste disposal practices of the HWHs are shown in Table-II. Around half of the HWHs (52.6%) labeled the bins of the waste according to their level of hazard. Only 17.9% disinfected the infected waste before disposal. Majority of them used disposable gloves (95.7%) and face masks (94.3%). One third had access to aprons while only 10.5% had access to protective shoes at their work place.

**Table-II:** Hospital Waste Disposal Practices of Hospital Waste Handlers (n=418)

Variable	F (%)
Labeling of Waste Bins	220 (52.6%)
Use of Danger Box for Sharps	345 (82.5%)
Syringe Needles Cut before Disposal	87 (20.9%)
High risk waste disposal area	
Open Area	55 (13.4%)
Community Bin	188 (45%)
Incineration	175 (41.9%)
Disinfection of the infected waste before disposal	75 (17.9%)
Use of Gloves	400 (96.7%)
Use of Masks	394 (94.3%)
Use of Aprons	154 (36.8%)
Use of Protective Shoes	44 (10.5%)

The frequency of hazards experienced by HWHs in the last six months is shown in Fig-I. Proportion of HWHs who experienced NSI and sharp injuries was 16.3% and 15.8% respectively. Around one tenths experienced skin symptoms (10%), eye symptoms (11.2%) and cough (9.1%) while 4.8% had difficulty in breathing. Fig.2 shows that among those who had either experienced NSI or sharp injury (n=117), 37.6% were screened for hepatitis B and C while 13.6% were screened for HIV AIDS.

**Fig.1:**
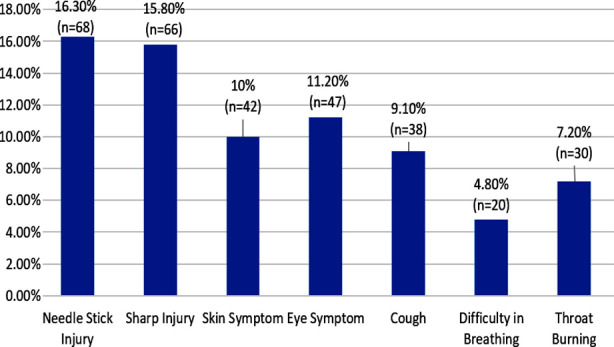
Frequency of hazards experienced in the last six months by HWHs due to exposure with hospital waste (n=418).

**Fig.2:**
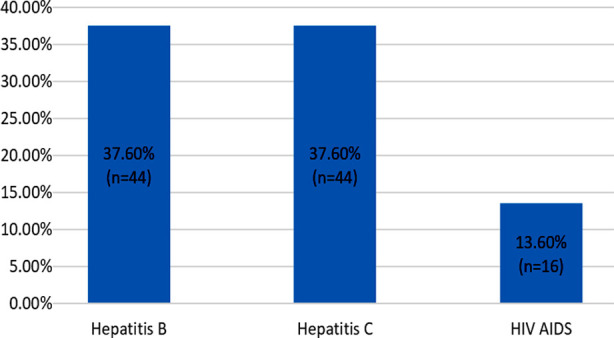
Screening Status of those HWHs who experienced needle stick or sharp injury (n=117).

Comparison of different hazards experienced in the last six months among different groups of HWHs is shown in Table-III. HWHs of private sector were significantly (p<0.001) less likely to experience NSI as compared to public sector while experience of sharp injuries was not significantly different. Occurrence of skin symptoms (p=0.023), cough (p<0.001) and difficulty in breathing (p=0.006) was also significantly lower in HWHs of private sector as compared to public sector hospitals. The proportion of HWHs experiencing difficulty in breathing was significantly higher (p=0.015) among the older age group of above 50 years as compared to younger age groups. Illiterate were significantly more likely to experience skin symptoms as compared to literate. The differences in proportions in the remaining results according to age groups, gender, educational status, work experience, placement area and status of training on waste disposal were not significantly different.

**Table-III:** Comparison of different hazards experienced in the last six months among different groups of HWHs.

	NSIs	Sharp Injury	Skin Symptom	Eye Symptom	Cough	Difficulty in Breathing
***Age***						
18 – 29 (n=134)	16.4% (22)	17.2% (23)	10.4% (14)	10.%( 14)	11.2% (15)	3.7% (5)
30 – 49 (n=228)	15.8% (36)	15.8% (36)	10.1% (23)	11.% (26)	7.9%( 18)	3.5% (8)
50 + (n=56)	17.9% (10)	12.5% (7)	8.9% (5)	10.7% (6)	8.9% (5)	12.5% (7)
	P=0.930	P=0.724	P=0.950	P=0.95	P=0.573	P=0.015
***Gender***						
Male (n=297)	16.2% (48)	15.5% (46)	10.8% (32)	11.8% (35)	8.4% (25)	3.7% (11)
Female (n=121)	16.5% (20)	16.5% (20)	8.3% (10)	9.1% (11)	10.7% (13)	7.4% (9)
	P=0.926	P=0.791	P=0.439	P=0.425	P=0.453	P=0.105
***Education***						
Literate (n=218)	18.8% (41)	13.7% (30)	5% (10)	8.5% (17)	6% (12)	5% (10)
Illiterate (n=200)	13.5% (27)	18.0% (36)	14.6% (32)	13.3% (29)	11.9% (26)	4.5% (10)
	P=0.321	P=0.065	P=0.004	P=0.271	P=0,104	P=0.390
***Work Experience***						
1-5 Years (n=245)	13.5% (33)	17.1% (42)	10.6% (26)	9.0% (22)	10.2% (25)	3.7% (9)
6 & above (n=173)	20.2% (35)	13.9% (24)	9.2% (16)	13.9% (24)	7.5% (13)	6.4% (11)
	P=0.065	P=0.367	P=0.648	P=0115	P=0.346	P=0.205
***Placement***						
Ward or ICU (n=287)	17.4% (50)	17.8% (51)	10.1% (29)	11.8% (34)	8.4% (24)	5.6% (16)
Other Area (n=131)	13.7% (18)	11.5% (15)	9.9% (13)	9.2%(12)	10.7% (14)	3.1% (4)
	P=0.344	P=0.100	P=0.955	P=0.416`	P=0.443	P=0.263
***Trained on HWM***						
Yes (n=184)	20.1% (37)	16.8% (31)	8.2% (15)	9.8% (18)	6.5% (12)	3.3% (6)
No (n=234)	13.2% (31)	15.0% (35)	11.5% (27)	12.0% (28)	11.1% (26)	6% (14)
	P=0.059	P=0.599	P=0.253	P=0.479	P=0.105	P=0.196
***Type of Hospital***						
Private (n=209)	8.6% (18)	14.4% (30)	6.7% (14)	9.1% ( 19)	3.8% (8)	1.9% (4)
Public (n=209)	23.9% (50)	17.2% (36)	13.4% (28)	12.9% (27)	14.4% (30)	7.7% (16)
	P<0.001	P=0.421	P=0.023	P=0.211	P<0.001	P=0.006

## DISCUSSION

The study found that exposure to occupational health hazards among HWHs is high and safety precautions adopted by them are inadequate. This is one of very few studies in Pakistan that has assessed occupational hazards of a vulnerable population of Health Waste Handlers in tertiary care hospitals of a mega city. The findings of the study provide useful insight about the state of occupational safety practices of HWHs in public sector hospitals which can help in identifying the measures needed to avoid health consequences associated with poor HWM. The study found that less than half of HWHs (44 %) had received some training regarding the methods of hospital waste disposal. This proportion is better than the findings of African studies in Ethiopia where proportion of trained HWHs was 18.6% and 31% respectively.^10^,^11^ However, it falls behind 83.7% reported in a study from Shiraz, Iran.^12^ This figure is very close to a study conducted in India which reports 44% of HWHs trained in HWM.^13^

The findings on waste disposal practices like labeling on bins (52.6%) and use of danger box for sharps (82.5%) was found better than the proportions reported by studies in other developing countries which include studies conducted in Iran and India.^12^,^14^ However, waste disinfection was markedly low at 17.9%. Disinfecting practices have also found to be low according to studies in other developing countries, the proportion of disinfecting the infected waste before disposal was found to be low in two Nigerian studies.^15^,^16^

Adherence to wearing PPEs like mask and gloves (>90%) was very high while practice of wearing gowns (36.8%) and protective shoes (10.5%) was found to be low although better than a previous study in a city of the India which reported < 3% workers using gown during working hours.^17^ The high use of PPE can be explained by the fact that this study was conducted during COVID pandemic and use of PPE increased remarkable post pandemic due to the media campaigns run on the television and other social networks.

The study also noted that the proportion of NSIs among HWH was found to be 16.3% and that of sharp injuries was 15.8% in the last six months. These proportions are remarkably lower than the frequencies reported in other developing countries. A study conducted in Institute of medical sciences India reported that 39.2% health care workers experienced NSIs in the last one year.^18^ Another study in Alexandria Egypt recorded NSIs to be 32.4%.^19^ Sharp injuries have also ranged from 30 to 38.3% in two studies conducted in Ethiopia.^10^,^20^ The low proportions of NSIs and Sharp injuries in this study can be explained by a shorter recall period of six months and better practices of wearing PPE post COVID era. Moreover, HWHs of the public sector experienced significantly higher NSIs as compared to the workers of the private sector which can be explained by better waste management practices in private sector.

An alarming finding of this study was that post injury, proportions for screening of Hepatitis B & Hepatitis C were quite low at 37.6 % while it was even lower at 13.6 % participants for the HIV Screening. It means about two third of the participants who were exposed to the injury were not screened for Hepatitis B and Hepatitis C while majority were not screened for HIV. On the contrary, a study conducted in the Dhaka City of Bangladesh reported 100 % screening in All HWH who had history of NSSI.^21^ This emphasizes the need of training of HCWs to improve their knowledge regarding NSIs as reported in a study conducted in Pakistan.^22^

The frequency of different symptoms due to occupational exposures was reported low in this study. Around one tenths reported experiencing skin problems, eye problems and cough while 7.4% reported throat burning and 4.8% experienced breathlessness. Other studies have reported higher proportions. A study of Gujrat India reported that the skin problems among sanitation workers were 30%.^17^ Lower frequencies experienced in this study can again be explained by shorter recall period and better preventive practices due COVID. Furthermore, these symptoms were experiences significantly less in private sector hospitals which strengthens the argument and HWM practices are better as compared to public sector hospitals.

### Limitations

It includes convenience sampling techniques adopted to include the participants which may have introduced selection bias. The study was conducted only in the single city and may not be representative of other cities in the province or big cities of other cities of Pakistan. There may be chance for memory bias because information was obtained regarding past working time period, however, to minimize memory bias, we kept a recall period of six months only. The study is also subject to response bias of HWHs.

## CONCLUSION

The HWM practices in tertiary care hospitals of Karachi is far from being satisfactory. The hospital waste management departments should follow the systematic guidelines of the World Health Organization for proper disposal of the hospital waste.

### Recommendations

Based on the findings of the study, we make a few recommendations. First, HWHs shall be trained on HWM and their knowledge and practices should be periodically assessed by hospital management. Second, they should be provided an enabling environment so that they can dispose of waste safely. Adequate resources should be provided to them which include colored bins to dispose of waste according to risk, disinfectants to neutralize infectious waste, incinerators to burn hazardous waste and appropriate personal protective equipment for handling waste. Third, screening of Hepatitis B and C and HIV shall be available to all HWHs who accidentally experience NSIs. Last, similar studies should be replicated at primary and secondary healthcare hospitals to understand the holistic magnitude of the problem. Prospective Interventional studies should be conducted in future to see which interventions work better in improving waste handling in the hospitals

### Authors Contribution:

**ED**: Conceived the study.

**ED, SS and UR**: Methodology was designed.

**ED, UR and DK**: Material preparation, data collection and analysis .

The first draft of the manuscript was prepared by and all authors who reviewed and suggested changes in the previous versions of manuscript.

All authors have read and approved the final manuscript.

All authors are responsible for the accuracy of work. Every Author made a contribution to the research study.
